# Surface Charge-Induced Scattering Enhancement of Diverse Dielectric Nanoscale Particles: A Simulation Study

**DOI:** 10.3390/nano15221738

**Published:** 2025-11-18

**Authors:** Siqi Zhang, Ang Li, Jiaan Wang, Linghao Wu, Siwen Gu, Xu Yang

**Affiliations:** 1 School of Photoelectric Engineering, Changzhou Institute of Technology, Changzhou 213032, China; 2 Office of Discipline Inspection, Changzhou Institute of Technology, Changzhou 213032, China; 3 The Higher Educational Key Laboratory for Measuring & Control Technology and Instrumentations of Heilongjiang Province, Harbin University of Science & Technology, Harbin 150080, China; yangxu@hrbust.edu.cn

**Keywords:** surface charge, scattering enhancement, dielectric nanoparticles, submicron particles, particle characterization

## Abstract

At the nanoscale, the scattered light intensity of particles significantly decreases and is easily affected by surface charges. However, under certain conditions, surface charges can induce a scattering enhancement effect, providing a new solution for the precise measurement of nanoparticles. Nevertheless, the universality of this effect in different material systems is still unclear. Therefore, we selected eight typical submicron dielectric particles encompassing oxides, polymers, semiconductors, and ceramics. Their optical responses under surface charging conditions were studied through numerical simulation. Results show that surface charges induce changes in the complex refractive index and significantly increase the scattering coefficient across all these particle types, compared to their neutral states. This enhancement effect is pronounced at the nanoscale particles, while at the submicron scale there is a clear critical size threshold, beyond which the enhancement effect significantly weakens. Surface charges also cause a spatial redistribution of scattered light intensity, enhancing the strength of forward, backward, and side scattering. These results confirm the cross-material universality of the surface charge-induced scattering enhancement effect. Our study provides a theoretical basis for extending optical measurement techniques for nanoscale particles and suggests considering surface charges in their detection and characterization to improve sensitivity and accuracy.

## 1. Introduction

The scattering signals of nanoscale particles are extremely weak and highly susceptible to interference from surface charges. This poses a severe challenge to the accurate measurement of their size and concentration. With the rapid development of fields such as environmental monitoring, biomedicine, and industrial manufacturing, the applications of such particles are becoming increasingly widespread. However, the bottleneck in measurement technology significantly restricts the in-depth development of these applications. In practice, most particles are not electrically neutral but carry surface charges due to friction, electric field effects, or discharge processes. This charged state invalidates the traditional Mie theory and its measurement methods, which assume electrical neutrality. Previous research shows that surface charges can enhance both forward and backward scattering by modifying the particles’ complex refractive index [1]. This effect provides a promising solution to current measurement limitations. Yet, existing studies are predominantly limited to single materials or ideal systems. A systematic investigation into how particles with diverse physicochemical properties respond to surface charges is still lacking. Specifically, the variation patterns of the optical characteristics of multiple particle categories in the charged state remain unclear.

The study of the optical behavior of charged particles is not only of great significance for particle measurement but also shows broad application prospects in fields such as remote sensing, meteorology, astronomy, and even deep space exploration. In remote sensing and meteorology, the main focus is on the scattering and attenuation effects of charged dust, cloud droplets, and other particles on microwaves and millimeter waves. For instance, the teams of Gao and Li systematically analyzed the interference mechanism of non-uniformly charged sand particles on electromagnetic wave propagation, providing a theoretical basis for communication attenuation in sandstorms [2,3,4,5,6,7]. In astronomical observations, Klačka and Kocifaj et al. discussed the influence of charged interstellar dust particles on near-field optics and radiative transfer, noting that even minute charges can significantly alter the scattering properties of particles [8,9,10,11,12,13,14,15]. Furthermore, in deep space exploration, the interaction between charged particles and electromagnetic waves is used to analyze the composition and distribution of planetary atmospheres and interstellar media [16,17]. These studies come from different fields, but they all jointly indicate that the optical properties of charged particles are a core issue across multiple disciplines. The advancements in theoretical research and practical measurement methods will have a driving effect on multiple cutting-edge scientific fields.

In the field of measurement science, our team has previously conducted a series of theoretical studies on the light scattering behavior of charged submicron particles. We first extended the complex refractive index model to charged particles [18] and established scattering calculation models suitable for both charged single particles and polydisperse particle systems [19]. These works defined the boundary conditions for the enhancement of forward and backward scattering by surface charges at the submicron scale. Furthermore, we numerically analyzed the light energy distribution of charged particles on the multi-ring photodetector of a laser particle size analyzer, providing a theoretical interface for practical measurements [1]. Experimentally, we have completed the qualitative verification of the charge-induced scattering enhancement effect, with a related manuscript currently under review. However, existing research has focused only on silica and polystyrene materials. A systematic comparison of the optical responses of different particle categories after charging has not been performed. In fact, material properties such as electrical conductivity, dielectric constant, and surface chemical characteristics may all influence the changes in the complex refractive index and scattering behavior after charging, thereby affecting the significance of the scattering enhancement effect.

Therefore, in order to systematically evaluate the universality and effective size range of the surface charge scattering enhancement effect in different dielectric materials, this paper selects eight typical particles from the fields of environmental protection, industry, medicine, and remote sensing. These particles cover the main dielectric material categories such as oxides, polymers, semiconductors, and ceramics. Through numerical simulation, this study focuses on investigating the changes in the complex refractive index, scattering coefficient, and angular scattered light intensity of these particles before and after charging. The aim is to verify whether the scattering enhancement effect universally exists across various material systems and to determine the critical size boundaries for significant enhancement in each material category. This research will provide key theoretical basis and screening criteria for extending charge-enhanced measurement techniques to multi-category nano and submicron particle systems.

## 2. Materials and Methods

### 2.1. Theoretical Model for Key Optical Parameters of Charged Particles

When excess charges exist on the surface of submicron particles, the number of electrons participating in the forced vibration within the light field increases. This leads to a change in the dielectric function of the particles, consequently affecting their complex refractive indexes. Based on the Lorentz model and the principle of dielectric function superposition, the extended complex refractive index for a charged spherical particle is given by Equation (1). The specific derivation process can be found in Equations (6)–(9) and (17) of Reference [18]. All simulations assume particles are in free space, approximating air as the surrounding medium (refractive index *m* = 1), to align with standard optical measurement configurations. The presented results are for particles carrying a negative surface charge, which is a common scenario for many dielectric materials.

(1) $$m_{i}={\left[\mu_{r}\varepsilon_{r}-\frac{4\mu_{r}e\rho_{s}}{D_{i}m_{e}\varepsilon_{0}\left(\omega^{2}+\gamma^{2}\right)}+i\frac{\mu_{r}(4e\rho_{s}\gamma +D_{i}m_{e}\sigma \omega^{2}+D_{i}m_{e}\sigma \gamma^{2})}{D_{i}m_{e}\varepsilon_{0}\omega \left(\omega^{2}+\gamma^{2}\right)}\right]}^{\frac{1}{2}}$$

where *m_i_* represents the complex refractive index of a particle with diameter *D_i_* carrying a surface charge (the subscript *i* indicates the particle size category, *i* = 1, 2, … K). *μ_r_* is the relative magnetic permeability. *ε*_0_ and *ε_r_* are the vacuum dielectric constant and the relative dielectric constant of the particle material, respectively, *ε*_0_ = 8.854 × 10^−12^ F/m. *σ* is the electrical conductivity, which is a parameter related to the particle material. *ω* represents the angular frequency of the incident light wave. *e* is the elementary charge, *e* = 1.602 × 10^−19^ C. *m_e_* is the mass of the elementary charge, *m_e_* = 9.109 × 10^−31^ kg. *γ* is the phenomenological damping constant.

The Mie scattering coefficient *Q_sca_*, absorption coefficient *Q_abs_*, and extinction coefficient *Q_ext_* for a single charged particle are given in Equation (2). The formula for calculating the angular scattered light intensity of charged particles is shown in Equation (3). These formulas are also applicable to charged polydisperse particle systems. However, as this study focuses on investigating the universality of scattering enhancement characteristics across different dielectric materials, analysis of polydisperse particle systems is not included.

(2) Qsca,i=2χ2∑n=1∞(2n+1)an,i2+bn,i2Qext,i=2χ2∑n=1∞(2n+1)Rean,i+bn,iQabs,i=Qext,i−Qsca,i

(3) $$I_{sca}=I_{0}\frac{\lambda^{2}}{8\pi^{2}{\bar{r}}^{2}}\sum_{i=1}^{K}N_{i}\left[{\left|S_{1,i}(\theta )\right|}^{2}+{\left|S_{2,i}(\theta )\right|}^{2}\right]$$

The scale parameter *χ_i_* of particles with diameter *D_i_* is defined as *χ_i_* = *πD_i_/λ*. *N_i_* represents the total number of particles with a diameter of *D_i_* in the measurement area and *W_i_* denotes the total weight of *N_i_* particles with *D_i_* (*i* = 1, 2, …, K). The relationship between *N_i_* and *W_i_* is given by $N_{i}=6W_{i}/(\rho \pi D_{i}^{3})$. *I*_0_ refers to the intensity of incident plane-polarized light, $\bar{r}$ represents the average distance from the particle system to the measuring point, *θ* denotes the scattering angle, and *φ* represents the azimuth angle. The calculation process for determining charged particle amplitude functions *S*_1,_*_i_*(*θ*) and *S*_2,_*_i_*(*θ*), as well as coefficients *a_n_*_,_*_i_* and *b_n_*_,_*_i_* involved in this calculation, can be found in Equations (1)–(9) [19], but will not be discussed further in this paper.

It is important to note that this model assumes a fixed surface charge density and a free-space environment. In practical scenarios, especially in liquid media, the surface charge is subject to dissipation mechanisms such as ionic screening and charge redistribution, which would modulate the observed enhancement effect. This study establishes the idealized upper limit of the phenomenon, providing a foundation for understanding charge-light interactions before introducing environmental complexities.

### 2.2. Selection and Description of Diverse Dielectric Particles

To systematically explore the universality of the scattering enhancement effect in different material systems, we select eight types of typical submicron particles from various application fields including environmental protection, industry, healthcare, and remote sensing as simulation objects. These materials exhibit broad representativeness in their physicochemical properties, covering major non-metallic material categories such as oxides, polymers, semiconductors, and ceramics. It should be noted that metal particles are excluded from this study. This is primarily because metal particles exhibit significant plasmon resonance characteristics, and their optical response mechanisms under external fields fundamentally differ from those of dielectric materials. The influence of surface charges on the scattering behavior of metal particles follows more complex patterns and requires investigation as an independent system. All eight selected material types are representative particles widely present in their respective fields with clear application backgrounds. The key parameters of these particles are listed in Table 1.

Oxide particles are materials widely found in the environment and commonly used in industries. This study selects silicon dioxide (SiO_2_) and titanium dioxide (TiO_2_) as representatives. SiO_2_ submicron particles serve as abrasives in chemical mechanical polishing within the semiconductor industry, carrier materials in drug delivery systems, and anti-reflection layers in optical films. The photocatalytic activity and refractive index of TiO_2_ are both at a relatively high level. Its nanoparticles are widely used in coatings, as ultraviolet shielding materials in sunscreens, and as photocatalysts in water treatment.

Polymer particles offer good modifiability and biocompatibility. This study selects polystyrene (PS) and polymethyl methacrylate (PMMA). PS microspheres are widely employed as model particles in both academia and industry due to their excellent monodispersity. Applications include colloidal self-assembly for photonic crystals, tracer particles for flow field visualization, and quality assessment of in vitro diagnostic reagents. PMMA exhibits excellent optical transparency. Its submicron particles are commonly used to fabricate functional units in microfluidic chips, optical diffuser plates, and substrate materials for biosensors.

Semiconductor particles possess unique photoelectric properties. This study selects zinc sulfide (ZnS) and cadmium sulfide (CdS). ZnS submicron particles are commonly used in the manufacture of cathode ray tubes and the phosphors of field emission display screens. They are also in great demand during the processes of preparing infrared window materials and making core–shell structure nanoparticles. CdS has a direct bandgap. Its quantum dots or submicron particles are widely used in solar cells, photodetectors, and biological fluorescence labeling, making it a typical subject for studying size-dependent photoelectric effects.

Ceramic particles possess characteristics such as high hardness, high temperature resistance and chemical inertness. This study selects aluminum oxide (Al_2_O_3_) and silicon nitride (Si_3_N_4_). Al_2_O_3_ submicron particles are important raw materials for engineering ceramics, used in manufacturing cutting tools, wear-resistant coatings, and observation windows for high-temperature furnaces. In environmental protection, they are also employed as efficient adsorbents for removing pollutants from water. Si_3_N_4_ submicron particles are key raw materials for manufacturing high-performance bearings, engine components, and high-temperature resistant structural ceramics. Due to its biocompatibility, it is also used in orthopedic implant materials.

It is noteworthy that the electrical conductivity of a material is highly sensitive to factors such as purity, crystallinity, microstructure, temperature, and the presence of impurities or dopants. This is particularly true for semiconductors and polymers, where the specific conductivity values of particles from different batches or sources can vary significantly, even by orders of magnitude. Therefore, during the simulation process, we only used conventional estimations and the typical or intrinsic conductivity range of this substance to roughly select a relatively reasonable value. This conductivity was not obtained by measuring the standard particulate matter of this substance.

## 3. Results and Analysis

To clarify the simulation conditions for the results presented herein, all optical responses were calculated for an incident light wavelength of 598 nm, assuming monochromatic and plane-polarized light consistent with a laser source. This wavelength was selected as it is commercially available, lies within the visible spectrum, and is suitable for characterizing the optical responses of all materials investigated, thereby ensuring the practical relevance of our findings. The following sections detail the universal trends observed under this condition. We note that in real measurement scenarios, such as particles on a substrate or in a matrix, the scattering will be influenced by the environment. Nevertheless, the core phenomenon of charge-induced scattering enhancement is expected to persist, though its quantitative details may vary. The following sections detail the universal trends observed under these specified conditions for negatively charged particles.

### 3.1. Universal Reduction in the Real Part of the Refractive Index

**Figure 1:**
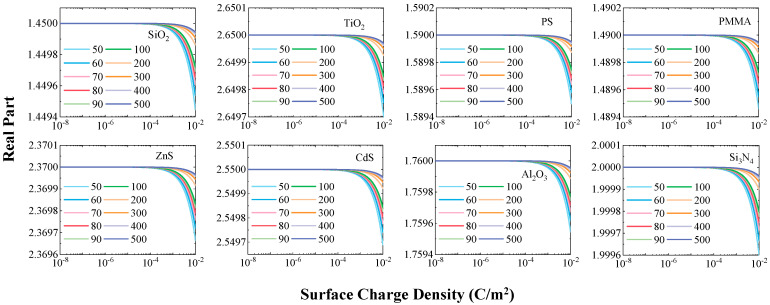
Variation in the real part of the complex refractive index with surface charge density (10^−8^ to 10^−2^ C/m^2^) for eight dielectric particle types.

Figure 1 shows the variation trend of the real part of the complex refractive index for eight types of typical submicron particles as the surface charge density increases from 1 × 10^−8^ C/m^2^ to 1 × 10^−2^ C/m^2^. Overall, the changes in the real part are consistent across all particles. When the surface charge density is below 1 × 10^−4^ C/m^2^, the real part remains essentially unchanged. Once the charge density exceeds this threshold, the real part begins to show a decreasing trend. This phenomenon is consistent with conclusions from previous studies on single-material particles, further verifying the universality of the influence of surface charges on the optical constants of particles.

It is noteworthy that the particle size significantly influences the magnitude of change in the real part. Smaller particles, such as the 50 nm silica particles which exhibited the most pronounced change, showed a decrease in the real part of approximately 0.4‰ under charge-saturated conditions. As the particle diameter increases, the change in the real part gradually weakens. This indicates that surface charges have a more significant impact on the dielectric behavior of smaller particles. This size dependence can be attributed to the increase in the ratio of charge distribution density on the particle surface to the particle volume as the size decreases, which enhances the modulation effect of the charges on electron vibration behavior.

**Figure 2:**
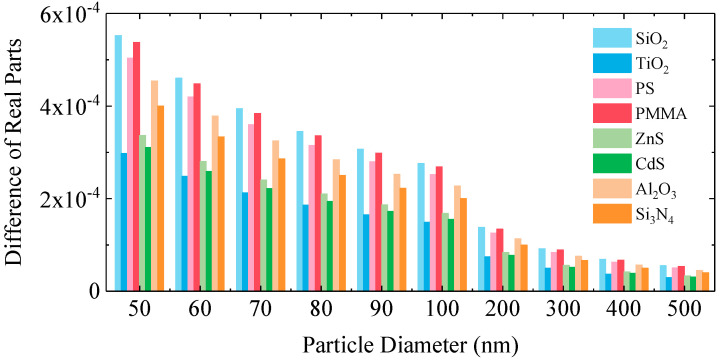
Difference in the real part of the refractive index between charge-saturated and electrically neutral states for particles of different diameters.

To further quantify the difference in the real part of the complex refractive index before and after charging, Figure 2 displays the difference in the real part between the electrically neutral state and the charge-saturated state for each particle. All observed differences in the real part remain below 0.0006. Although the magnitude of change is limited, its cumulative effect on scattering behavior cannot be ignored at the submicron scale. Furthermore, no significant categorical differences are observed between different materials. This indicates a high consistency in the trend of real part variation across all particle types, including oxides, polymers, semiconductors, and ceramics. For instance, materials with initially similar real parts, such as SiO_2_ and Al_2_O_3_, exhibit comparable magnitudes of change after charging. This demonstrates that, within the material range selected for this study, the response of the real part of the complex refractive index to surface charge is primarily governed by particle size, with the intrinsic optical properties of the materials themselves having minimal influence.

### 3.2. Material-Dependent Increase in the Imaginary Part

**Figure 3:**
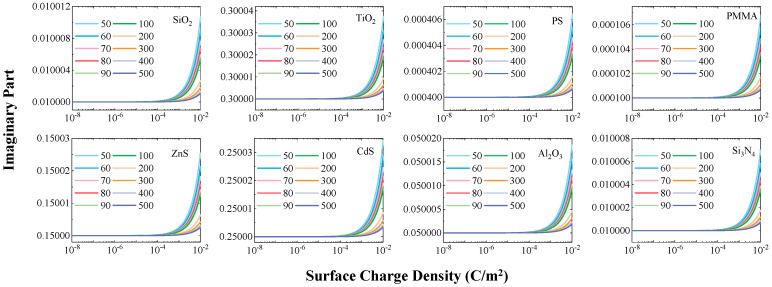
Variation in the imaginary part of the complex refractive index with surface charge density (10^−8^ to 10^−2^ C/m^2^) for the eight particle types.

Figure 3 illustrates the evolution of the imaginary part of the complex refractive index for the eight particle types with increasing surface charge density. The imaginary part of the refractive index exhibits a similar charge density threshold (~1 × 10^−4^ C/m^2^) for change as the real part. However, beyond this threshold, its behavior diverges, showing a systematic increase that signifies enhanced energy dissipation. This phenomenon indicates that surface charges enhance the particle’s ability to dissipate light energy, corresponding to a change in the absorption characteristics. Similarly, the change in the imaginary part is more pronounced for smaller particles, further confirming the enhanced charge effect at the nanoscale.

**Figure 4:**
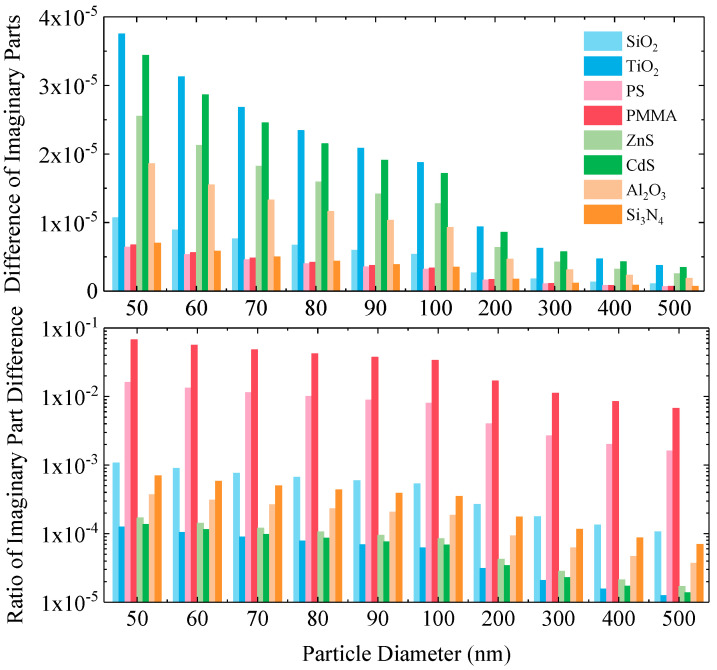
(**Upper**) Absolute and (**Lower**) relative change in the imaginary part of the refractive index after charge saturation for different materials and particle sizes.

To more precisely evaluate the relative magnitude of the change in the imaginary part, the upper section of Figure 4 presents the absolute difference in the imaginary part before and after charging, while the lower section shows the ratio of this difference to the initial imaginary part value. Analysis of the absolute differences reveals that the increase in the imaginary part for all particles is within 0.00004 (a.u.), which is comparable in order of magnitude to the change observed in the real part. However, significant differences emerge between different materials when considering the relative change ratio.

Specifically, polymer particles such as PMMA and PS exhibit the highest proportional increase in their imaginary part. For example, the imaginary part of 50 nm PMMA particles increases by approximately 6.74% after charge saturation. In comparison, materials like SiO_2_, Al_2_O_3_, and Si_3_N_4_ show moderate increases in their imaginary parts, while semiconductor particles such as TiO_2_, ZnS, and CdS are minimally affected by the charge, displaying only weak relative changes. This difference indicates that the intrinsic value of the material’s imaginary part is a key factor determining its sensitivity to surface charges. Insulator materials with lower initial imaginary parts and smaller dielectric losses exhibit more easily amplified relative changes.

Consistent with the behavior observed for the real part, the change in the imaginary part also exhibits a clear size dependence. As the particle diameter increases, both the absolute difference and the relative change ratio of the imaginary part gradually decrease. This again verifies that surface charges have a more pronounced effect on the optical constants of smaller particles.

### 3.3. Three-Stage Evolution and Critical Size of Scattering Coefficient Enhancement

**Figure 5:**
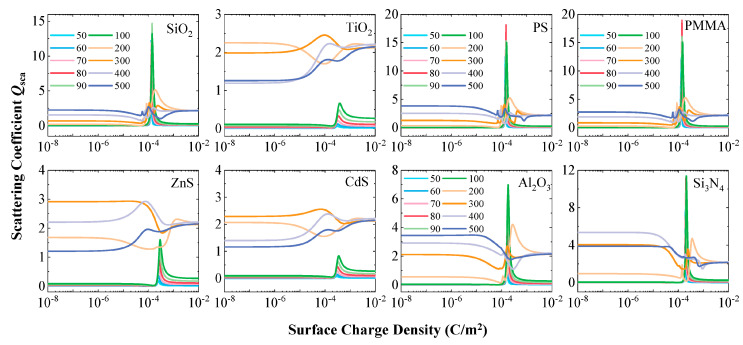
Evolution of the scattering coefficient (*Q*_sca_) as a function of surface charge density for the eight dielectric particle types.

Figure 5 systematically displays the evolution of the scattering coefficient *Q*_sca_ for eight representative submicron particle types with increasing surface charge density. Overall observation reveals that the changes in *Q*_sca_ for all particles exhibit a highly consistent three-stage characteristic. When the surface charge density *ρ*_s_ is below 1 × 10^−5^ C/m^2^, the *Q*_sca_ of each particle remains stable, identical to the electrically neutral state. As the charge density enters the range of 1 × 10^−5^ to 1 × 10^−3^ C/m^2^, *Q*_sca_ begins to show significant fluctuations, indicating that the optical properties of the particles enter a sensitive transition region within this charge density range. Notably, when the charge density exceeds 1 × 10^−3^ C/m^2^, the *Q*_sca_ of each particle gradually converges to a new stable state. This stable value is generally higher than that in the electrically neutral state, providing a theoretical basis for utilizing surface charges to enhance scattering signals.

A deeper analysis of the oscillation phase reveals significant differences in the response intensity to charges among different materials. Nanoparticles of SiO_2_, PS, PMMA, Al_2_O_3_, and Si_3_N_4_ exhibit strong fluctuations in *Q*_sca_ around *ρ*_s_ ≈ 1 × 10^−4^ C/m^2^, while the three materials TiO_2_, ZnS, and CdS show relatively smaller fluctuation amplitudes at the nanoscale. This difference may be related to the intrinsic electrical properties of the materials, suggesting that high-conductivity materials might respond to surface charges through different mechanisms.

**Figure 6:**
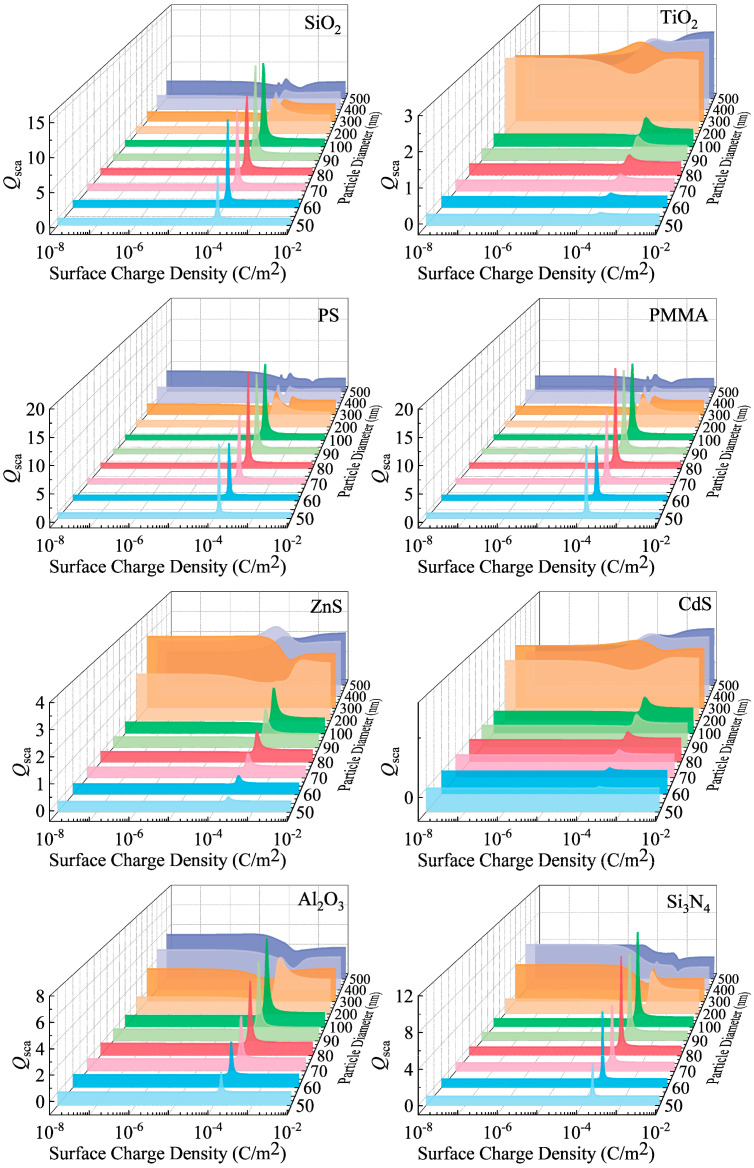
Three-dimensional representation of the scattering coefficient (*Q*_sca_) variation with surface charge density and particle diameter.

To more comprehensively reveal the variation pattern of *Q*_sca_ with particle size, Figure 6 provides a three-dimensional representation of the changes in the scattering coefficient. This perspective uncovers an important phenomenon: the fluctuation amplitude of *Q*_sca_ does not increase monotonically with decreasing particle size. Instead, it reaches a maximum near the boundary between the nanoscale and submicron scale (approximately 100 nm). This finding revises the conventional understanding that the charge effect is simply more pronounced in smaller particles. It indicates that particles within this specific size range exhibit particular sensitivity to surface charges.

From an application perspective, Table 2 summarizes the critical size boundaries for the onset of the scattering enhancement effect in each material. The data show that the *Q*_sca_ of nanoscale particles (*d* < 100 nm) from all eight material types is significantly higher under saturated charging compared to their neutral states, confirming the universality of the surface charge-induced scattering enhancement effect at the nanoscale. However, the effective enhancement size range in the submicron region varies among materials. The enhancement boundary extends to 500 nm for SiO_2_, PMMA, and Al_2_O_3_, while it is 300 nm for TiO_2_, CdS, and Si_3_N_4_. PS and ZnS exhibit intermediate values. These material-dependent differences provide a reference for optimizing charge-enhanced measurement strategies for different particle systems.

The observed enhancement in the scattering cross-section is a direct consequence of the surface charge induced modification of the complex refractive index, as shown in Equation (1). This mechanism is fundamentally a broadband effect, arising from the perturbation of the material’s dielectric function by free charges, and is not contingent upon specific electronic or vibrational absorption resonances. Consequently, the scattering enhancement effect is applicable across a wide spectral range, including visible light spectroscopy techniques such as Raman scattering.

### 3.4. Spatial Redistribution of Scattered Light Intensity

**Figure 7:**
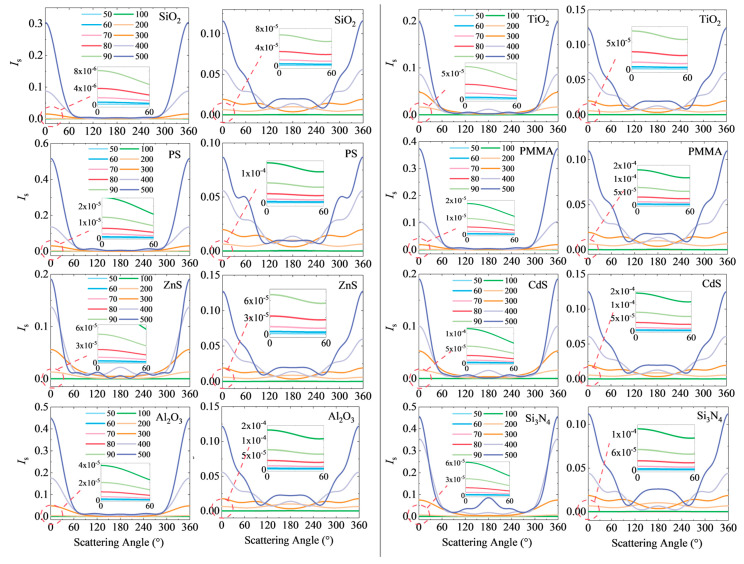
Angular distribution of scattered light intensity comparing electrically neutral state and charged (*ρ*_s_ = 1 × 10^−3^ C/m^2^) states for representative particles.

Figure 7 systematically displays the distribution changes in angular scattered light intensity for eight representative submicron particle types before and after charging. The left side of the figure shows the scattered light intensity distribution in the electrically neutral state, while the right side shows the distribution when the particles carry a surface charge density of *ρ*_s_ = 1 × 10^−3^ C/m^−2^. Through comparative analysis, it can be clearly observed that surface charge exerts a significant influence on the spatial redistribution of scattered light intensity.

In the forward scattering direction (near 0°), all nanoscale particles (*d* ≤ 100 nm) exhibit a significant enhancement in scattering after charging. However, for submicron particles, a distinct size boundary determines whether forward scattering is enhanced. When the particle diameter is below this critical size, forward scattering is enhanced; conversely, when exceeding this size, forward scattering becomes suppressed. This phenomenon aligns with the previously described variations in scattering coefficients, further confirming that the regulatory effect of surface charges on forward scattering exhibits significant size dependence.

In the backward scattering direction (near 180°), a more universal enhancement phenomenon is observed. Except for a few particles with larger diameters, nearly all studied particles exhibit a significant increase in backward scattering intensity after charging. This indicates that backward scattering is more sensitive to surface charges and is relatively less influenced by particle type and size. It may therefore serve as a more reliable detection channel for charge-enhanced measurements.

In the side-scattering angular range (90° to 150°), the scattered light intensity distribution of charged particles exhibits more distinct angular features. Compared to the electrically neutral state, the differences in scattering signals within this angular range are more pronounced for charged particles. This provides new possibilities for the joint inversion of both particle size and surface charge density through multi-angle scattering measurements.

**Figure 8:**
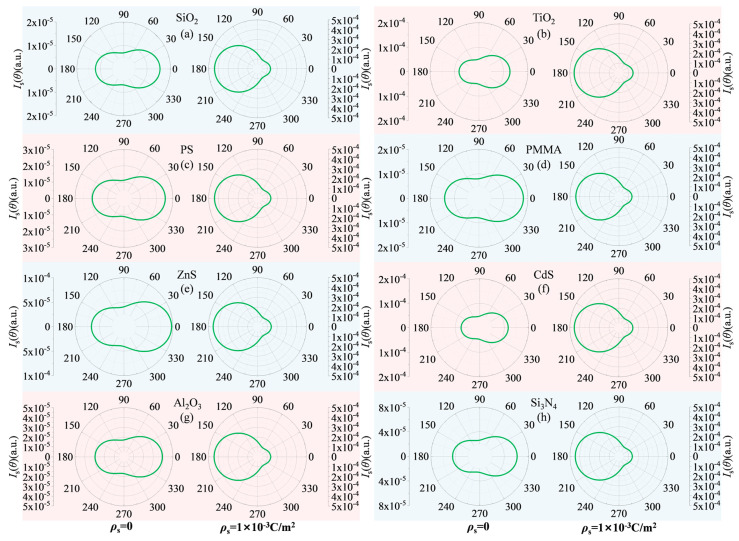
Polar plots comparing the scattered light intensity distribution for 100 nm particles between (**left**) electrically neutral state and (**right**) charge-saturated states.

To more intuitively display the spatial redistribution of scattered light intensity, Figure 8 presents polar scatter plots for particles of different properties with a diameter of 100 nm, comparing their states before and after charging. In the electrically neutral state, the scattered light intensity of all particles is predominantly forward-directed, with energy flux distribution exhibiting a characteristic peanut shape. Under the influence of surface charge, the scattered light intensity not only increases significantly in total magnitude but also undergoes a fundamental transformation in its spatial distribution pattern: the proportion of forward scattering decreases relatively, while the proportions of backward and side-scattering increase substantially. This redistribution of energy flow results in a fuller scattering profile in the polar plots, particularly in the backward hemisphere where the intensity enhancement can reach several orders of magnitude, as evidenced by the coordinate axis values.

It is noteworthy that although the initial scattering distributions of particles from different materials differ in the electrically neutral state, they all exhibit the aforementioned consistent scattering redistribution trend under the modulation of surface charges. This indicates that the surface charge-induced scattering enhancement and spatial redistribution phenomena possess universality across materials and systems. The underlying physical mechanism primarily originates from the modulation of the overall dielectric response of the particles by the charges, rather than depending on specific material chemical properties.

### 3.5. Absorption Suppression and Stable Extinction

**Figure 9:**
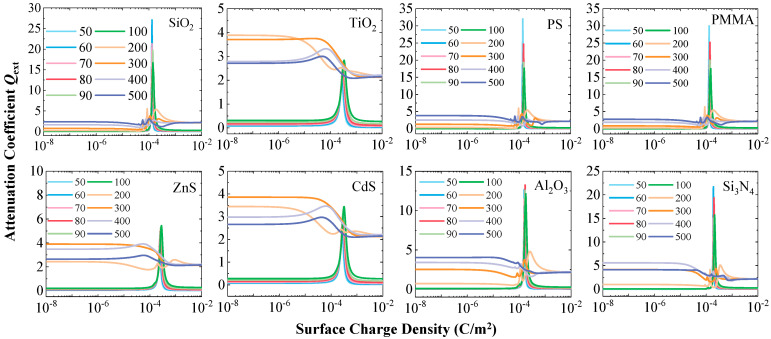
Variation in the absorption coefficient (*Q*_abs_) with surface charge density for the eight particle types.

**Figure 10:**
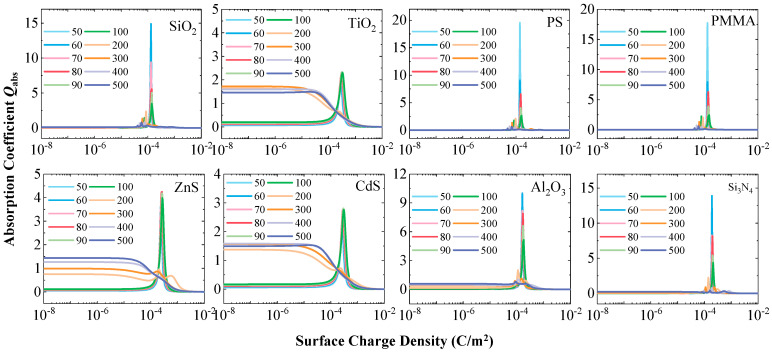
Variation in the extinction coefficient (*Q*_ext_) with surface charge density for the eight particle types.

As an important supplement to the scattering characteristics, Figure 9 and Figure 10, respectively, show the evolution of the absorption coefficient and the extinction coefficient for the eight particle types during the charging process. Analysis reveals that when the surface charge density is in the range of 1 × 10^−5^ C/m^2^ to 1 × 10^−3^ C/m^2^, the *Q*_abs_ of all particles fluctuates to varying degrees. Particularly noteworthy is that after the charge reaches saturation, the absorption coefficient stabilizes at a level lower than that in the electrically neutral state. This phenomenon clearly indicates that the presence of surface charges suppresses the particle’s absorption of light energy to some extent. This absorption suppression effect is more pronounced for larger particles of materials with strong intrinsic absorption, such as TiO_2_, ZnS, and CdS.

Although surface charges significantly enhance the particle scattering capability while simultaneously reducing their absorption, Figure 10 shows that the overall extinction coefficient *Q*_ext_ of the particles does not undergo drastic changes before and after charging. This indicates that the magnitude of scattering enhancement is approximately balanced by the degree of absorption reduction, maintaining the total attenuation capability of the particles for incident light at a relatively stable level.

This finding holds significant practical implications. When conducting optical measurements on charged particles, the total attenuation signal detected by the instrument may be similar to that of traditional electrically neutral particles. But the underlying physical mechanism has undergone a fundamental change, shifting from being dominated by scattering and absorption to being dominated solely by scattering. Therefore, only relying on traditional inversion algorithms based on electrically neutral state models would be not sufficient to accurately determining the true state of the particles. The compensatory relationship between absorption suppression and scattering enhancement revealed in this study provides a crucial theoretical basis for developing specialized optical measurement techniques suitable for charged particles.

## 4. Conclusions

This study, through systematic simulation, reveals the universal modulation laws of surface charges on the optical properties of multi-category nano and submicron particles. The results show that surface charges universally alter the complex refractive index of all particles, namely, causing a universal reduction in the real part and a concurrent rise in the imaginary part. This effect is particularly pronounced at the nanoscale. Within a specific charge density range, the scattering coefficient exhibits characteristic oscillations and ultimately stabilizes in an enhanced state after charge saturation, with the strongest enhancement sensitivity observed at the nanoscale-submicron boundary. Furthermore, the increase in charge not only enhances the overall scattering intensity but also causes the spatial redistribution of the scattering energy of all particles. Specifically, forward scattering shows size-selective enhancement, while backward scattering demonstrates a universal enhancement characteristic across material systems. Simultaneously, the resolution capability of side scattering is significantly improved. Additionally, the study observes that while enhancing scattering, the charges suppress the absorption capacity, resulting in relatively stable extinction characteristics. This indicates that the interaction mechanism between charged particles and light has shifted from being dominated by both scattering and absorption to being dominated solely by scattering. Experimental validation of this scattering enhancement effect for selected materials, such as silica and polystyrene, has been conducted and supports the simulation predictions, with details reported in a separate manuscript under review. These findings not only deepen the understanding of the interaction mechanism between light and charge but, more importantly, provide a new approach for extending optical measurement techniques for micro-nano particles. By actively utilizing the scattering enhancement effect of surface charges, the sensitivity limitations of traditional optical measurement techniques can be effectively overcome, establishing a solid theoretical foundation for achieving precise, multi-parameter measurement of nanoscale particles. This study, employing spherical particles with uniform charge distribution, establishes a fundamental framework. Future work will utilize advanced numerical methods to extend this model to more complex scenarios, including non-spherical particles, surface roughness, charge inhomogeneity, polydisperse systems, and core–shell structures.

## Figures and Tables

**Table 1:** Key material parameters of the diverse dielectric particles.

Category	Particle Name	Abbreviation	Complex Refractive Index (Electrically Neutral State)	Selected Electrical Conductivity (S/cm)
Oxide	Silicon Dioxide	SiO_2_	1.45 + 0.01*i*	1 × 10^−12^
	Titanium Dioxide	TiO_2_	2.65 + 0.3*i*	1 × 10^−5^
Polymer	Polystyrene	PS	1.59 + 0.0004*i*	1 × 10^−14^
	Polymethyl Methacrylate	PMMA	1.49 + 0.0001*i*	1 × 10^−14^
Semiconductor	Zinc Sulfide	ZnS	2.37 + 0.15*i*	1 × 10^−3^
	Cadmium Sulfide	CdS	2.55 + 0.25*i*	1 × 10^−2^
Ceramic	Aluminum Oxide	Al_2_O_3_	1.76 + 0.05*i*	1 × 10^−12^
	Silicon Nitride	Si_3_N_4_	2.0 + 0.01*i*	1 × 10^−14^

**Table 2:** Critical particle diameter for significant scattering enhancement upon charge saturation for each material.

Name	SiO_2_	TiO_2_	PS	PMMA	ZnS	CdS	Al_2_O_3_	Si_3_N_4_
Diameter (nm)	*d* < 500	*d* < 200	*d* < 400	*d* < 500	*d* < 300	*d* < 300	*d* < 400	*d* < 300

## Data Availability

We did not generate any datasets because our work was conducted using a theoretical and mathematical approach.
